# MSC-Derived Small Extracellular Vesicles Attenuate Autoimmune Dacryoadenitis by Promoting M2 Macrophage Polarization and Inducing Tregs *via* miR-100-5p

**DOI:** 10.3389/fimmu.2022.888949

**Published:** 2022-07-06

**Authors:** Na Li, Zhiqi Gao, Lu Zhao, Bei Du, Binyun Ma, Hong Nian, Ruihua Wei

**Affiliations:** ^1^ Tianjin Key Laboratory of Retinal Functions and Diseases, Tianjin Branch of National Clinical Research Center for Ocular Disease, Eye Institute and School of Optometry, Tianjin Medical University Eye Hospital, Tianjin, China; ^2^ Department of Medicine/Hematology, Keck School of Medicine of the University of Southern California, Los Angeles, CA, United States

**Keywords:** small extracellular vesicles, human umbilical cord mesenchymal stem cells, autoimmune dacryoadenitis, tregs, macrophages, miR-100-5p

## Abstract

**Background:**

Mesenchymal stem cell-derived small extracellular vesicles (MSC-sEVs) have been increasingly proved as promising immunomodulators against some autoimmune disorders. However, the possible effect and the underlying mechanism of MSC-sEVs in autoimmune dry eye have been rarely studied.

**Methods:**

Small extracellular vesicles from human umbilical cord mesenchymal stem cells (hUC-MSC-sEVs) were subconjunctivally injected to rabbit dry eye model, and their preventive or therapeutical effects were assessed by recording the clinical and histological scores. Quantitative real-time PCR (Q-PCR), western blot and flow cytometry were performed to evaluate the immunomodulatory effects of hUC-MSC-sEVs on macrophages and T regulatory cells (Tregs) both *in vivo* and *in vitro*, and the *in vitro* T cell proliferation was detected by Bromodeoxyuridine (BrdU) assay. In addition, high expression of miR-100-5p in hUC-MSC-sEVs was identified by Q-PCR, and the functional role of sEVs-miR-100-5p on macrophages was explored by a series of co-culture experiments using sEVs derived from hUC-MSCs transfected with miR-100-5p inhibitor.

**Results:**

We firstly demonstrated that hUC-MSC-sEVs had the preventive and therapeutical effects on rabbit autoimmune dacryoadenitis, an animal model of Sjögren’s syndrome (SS) dry eye. Further investigation revealed that hUC-MSC-sEVs administration effectively elicited macrophages into an anti-inflammatory M2 phenotype and elevated the proportion of Tregs both *in vivo* and *in vitro*, which contributed to reduced inflammation and improved tissue damage. Importantly, hUC-MSC-sEVs-educated macrophages with M2-like phenotype exhibited strong capacity to inhibit CD4+ T cell proliferation and promote Treg generation *in vitro*. Mechanistically, miR-100-5p was highly enriched in hUC-MSC-sEVs, and knockdown of miR-100-5p in hUC-MSC-sEVs partially blunted the promotion of hUC-MSC-sEVs on M2 macrophage polarization and even attenuated the effect of hUC-MSC-sEVs-educated macrophages on T cell suppression and Treg expansion.

**Conclusion:**

Our data indicated that hUC-MSC-sEVs alleviated autoimmune dacryoadenitis by promoting M2 macrophage polarization and Treg generation possibly through shuttling miR-100-5p. This study sheds new light on the application of MSC-sEVs as a promising therapeutic method for SS dry eye.

## Introduction

Sjögren’s syndrome (SS) dry eye is an intractable autoimmune disorder, characterized by lymphoid and myeloid cell infiltration of the lacrimal glands (LGs) and subsequent tissue damage, reflected clinically by ocular discomfort and even visual loss (1–3). Although the etiology of SS remains ill defined, available evidence have pointed out that macrophages play an important role both during the onset and resolution of inflammation in SS dry eye (4–6).

In response to environmental signals, macrophages can differentiate into the classical pro-inflammatory M1 phenotype or alternative anti-inflammatory M2 phenotype. Unbalanced M1/M2 polarization is now considered to be critical in the pathogenesis of chronic inflammatory and autoimmune diseases (7). A prolonged activation of M1 macrophages could drive inflammation (8), and inflammatory M1 activation has been observed in the conjunctiva and cornea of murine dry eye induced by desiccation stress (9, 10). However, M2 macrophages are involved in anti-inflammation and tissue repair (11). Decreased number of M2-like macrophages is associated with increased inflammatory grading of the salivary gland lesions in SS patients (12). Besides, our team has previously demonstrated a defective M2 macrophage activation in rabbit autoimmune dacryoadenitis, an animal model which closely mimics human SS dry eye (6), indicating the beneficial effects of M2 macrophages in SS dry eye. Furthermore, M2 macrophages can be considered as one kind of regulatory cells in the innate immunity, and are important in inducing regulatory T cells (Tregs) with immunosuppressive roles (13). Tregs can prevent lacrimal gland autoimmunity (14), and transient partial depletion of Tregs in female NOD mice is sufficient to drive dacryoadenitis (15).

Accumulating data from preclinical studies indicate that the beneficial effects of mesenchymal stem cells (MSCs) were largely ascribed to the paracrine mediators containing small extracellular vesicles (sEVs) (16, 17), and MSC-sEVs have been recently shown to be effective in treating various inflammatory and autoimmune diseases, such as colitis and rheumatoid arthritis (18–20). MicroRNAs (miRNAs) which can regulate gene expression at post-transcriptional level make up an important fraction of MSC-sEVs, and are crucial in MSC-sEVs-mediated immunosuppressive effects (19). Recent studies have shown that MSC-sEVs can regulate the biological activities of immune cells, including macrophages and T cells through transferring miRNAs (21). For example, sEVs from IL-1β-primed human umbilical cord mesenchymal stem cells (hUC-MSCs) ameliorated the symptoms of murine sepsis partially because of transferred sEVs-miR-146a induced M2 macrophage polarization (22). However, currently the role and the underlying mechanism of sEVs derived from hUC-MSCs (hUC-MSC-sEVs) in SS dry eye remain unknown.

In this study, we investigated the protective effects of hUC-MSC-sEVs administration in rabbit SS dry eye model and explored its immunomodulatory mechanism, especially its role on macrophage polarization. We found that hUC-MSC-sEVs administration efficiently alleviated the development of rabbit autoimmune dacryoadenitis, and hUC-MSC-sEVs could induce M2 macrophage polarization and increase the proportion of Tregs both *in vivo* and *in vitro*. Furthermore, hUC-MSC-sEVs-educated macrophages were able to inhibit the proliferation of CD4+ T cells and promote the generation of Tregs, and miR-100-5p capsuled in hUC-MSC-sEVs contributed a lot to the regulation of hUC-MSC-sEVs on macrophage phenotype and function.

## Materials and Methods

### Approval for Experiments Using Rabbit and Human Samples

Adult female New Zealand white rabbits (3.5-4 kg) were obtained from Vital River Laboratory Animal Technology (Beijing, China). All procedures with animals were performed in accordance with the guidelines of the Laboratory Animal Care and Use Committee of Tianjin Medical University Eye Hospital and the ARVO Statement for the Use of Animals in Ophthalmic and Vision Research (Permit Number: TJYY20181217001). The collection and use of human blood samples and human umbilical cords were approved by the Ethics Committee of Tianjin Medical University Eye Hospital (Permit Number: 2018KY(L)-41).

### Isolation and Identification of sEVs

Fresh human umbilical cords were obtained and digested to isolate hUC-MSCs as previously described in our work (6). Briefly, the umbilical cords were washed twice, cut into around 1 mm^3^ pieces and then digested with 0.1% collagenase type II (Gibco, USA) for 1h on shaker at 37°C. After that, the cell suspension was filtered through meshes and then centrifuged. The harvested pellets were resuspended in complete culture medium and subsequently incubated at 37°C. The medium with 10% fetal bovine serum (FBS, cat# 16000-044, Gibco, USA) was refreshed every 2-3 days. The phenotype of hUC-MSCs was determined by flow cytometry using specific antibodies as follows: CD29-FITC (clone TS2/16), CD34-FITC (clone 4H11), CD44-FITC (clone IM7), CD45-FITC (clone HI30) and CD105-PE (clone SN6) (eBioscience™, USA); CD73-FITC (clone AD2), CD90-FITC (clone 5E10), CD11b-FITC (clone M1/70) and HLA-DR-PE (clone LN3) (Biolegend, USA). The abilities of hUC-MSCs to differentiate into adipocytes, osteoblasts and chondrocytes were evaluated as described previously (23).

Passage 3-5 hUC-MSCs (2-2.5×10^6^ cells) were cultured with 25ml culture medium containing 10% sEVs-free FBS (prepared by overnight ultracentrifugation at 110,000 g at 4°C) in 175 cm^2^ cell culture flask for 48h, and at least 400ml culture medium was collected for one batch of cultures. sEVs were isolated using differential centrifugation based on previously described methods (24). In brief, 400ml supernatants collected from cultured MSCs were centrifuged at 2000 ×g for 10 min followed by 10000 ×g for 30 min to remove cellular debris and dead cells, and then at 110000 ×g for 70 min twice *via* ultracentrifugation (Type 45Ti rotor, Beckman Coulter, USA), all at 4°C. Then the pellets were resuspended in 400-500μl PBS, sterilized by filtration through a prerinsed 0.22-μm filter and stored at -80°C until use. BCA protein assay reagent kit (Solarbio, China) was used to detect the protein concentration of the collected sEVs, and nanoparticle tracking analysis (NTA) (NanoSight NS300, UK) was performed to determine the diameter and particle concentration of sEVs. The mean protein and particle contents of sEVs were 0.55 ± 0.05 μg/μl and 5.72×10^8^ ± 3.12×10^7^ particles/μl. Visualization of sEVs was assessed by HT7700 transmission electron microscope (TEM) (HITACHI, Japan). 20 μg sEVs were denatured in SDS loading buffer at 95°C for 5 min, and then were used for western blot assays to detect the typical sEV markers including CD9, CD63, CD81 and tumor susceptibility 101 (TSG101), and the non-sEV marker Calnexin.

### Autoimmune Dacryoadenitis Induction and Treatment

Autoimmune dacryoadenitis was induced as described previously (25). Briefly, the epithelial cells purified from normal rabbit LGs (pLGECs) were irradiated and then co-cultured with autologous peripheral blood lymphocytes (PBLs) at the ratio of 1:1. After 5 days, the activated PBLs from mixed cell reactions were harvested and adoptively injected back into rabbits *via* ear margin vein to induce autoimmune dacryoadenitis.

To explore the preventive and therapeutic effects of hUC-MSC-sEVs, two administration schedules were arranged: 1) Model rabbits in the preventive experiments were subconjunctivally injected with hUC-MSC-sEVs (30 μg) (hUC-MSC-sEVs group, n=9) or PBS (untreated group, n=9) at the early stage of disease after adoptive transfer of activated PBMCs (days 1, 3, 5, 7 and 9). Normal rabbits were used as controls (normal group, n=9). 2) Model rabbits in the therapeutic experiments were treated at the developed stage (2 weeks after transfer) with every-two-day injection of hUC-MSC-sEVs (30 μg) (hUC-MSC-sEVs group, n=6) or PBS (untreated group, n=6) for 5 doses. Based on our preliminary experiments, this administration protocol had been confirmed to be most effective for rabbit autoimmune dacryoadenitis.

### Clinical and Histopathological Assessment

The clinical assessments were conducted every 2 weeks after the first hUC-MSC-sEVs administration. The tear break-up time (BUT), the severity of the corneal fluorescein staining and the tear production were detected and recorded as described previously (26). Histopathological evaluation of LGs and conjunctivas was performed by hematoxylin and eosin (H&E) staining at the end of the experiment (8 weeks after transfer), and the number of focus (aggregate of >50 lymphocytes) per 4 mm^2^ in LGs was recorded as previously reported (26).

### THP-1 Cell Culture and Treatment

The human monocytic cell line THP-1 was obtained from Stem Cell Bank, Chinese Academy of Sciences, and was differentiated into macrophages by treatment with 160 ng/ml phorbol 12-myristate 13-acetate (PMA, Sigma Aldrich, USA) for 24h. Then the differentiated macrophages (5×10^5^ cells/ml) were stimulated with 100 ng/ml lipopolysaccharides (LPS) from E. coli K12 (ultrapure, cat# tlrl-peklps, Invivogen, USA) and 50 ng/ml recombinant human interferon-γ (IFN-γ, R&D Systems, USA) for an additional 24 hours. After that, the adherent cells were incubated with fresh medium containing hUC-MSC-sEVs (5 μg/ml) or PBS for 48 h. The treated macrophages were finally collected for the following co-culture experiment or RNA and protein harvest.

### Transfection of miR-100-5p Mimics and Inhibitors

miR-100-5p mimics and negative-control (NC) mimics, as well as miR-100-5p inhibitor and NC inhibitor, were designed and synthesized by Gene Pharma (Suzhou, China). LPS+IFN-γ-stimulated macrophages were transfected with miR-100-5p mimics (100 nM) or NC mimics using Lipofectamine 2000 Reagent (Thermo Fisher Scientific, USA) according to the manufacturer’s instructions. After 48h transfection, the cells were collected for subsequent experiments. hUC-MSCs were transfected with 100 nM of miR-100-5p inhibitor, NC inhibitor, miR-100-5p mimics and NC mimics for 6h. Then the culture medium was replaced with that containing 10% sEVs-free FBS for sEVs isolation. The effect of transfection was confirmed by Q-PCR.

### Labeling and Internalization Assay of sEVs

To identify the internalization of hUC-MSC-sEVs into macrophages, hUC-MSC-sEVs were stained with PKH26 fluorescent dye (Sigma-Aldrich, USA) according to the manufacturer’s instructions and centrifuged again to remove contaminating dye. Then, the PKH26-labeled hUC-MSC-sEVs (10 μg/ml) were co-cultured with PMA-treated macrophages. After 6h, the macrophages were washed with PBS and fixed in 4% paraformaldehyde for 15min. Thereafter, the nucleus was stained with DAPI (Solarbio, China). Finally, the cells were observed and photographed with the laser scanning confocal microscope (LSM800, Zeiss, Germany).

### CD4+ T Cell Preparation and Co-Culture

CD4+ T cells were isolated from the healthy human peripheral blood by magnetic activated cell sorting (MACS) (negative selection), using the CD4+ isolation Kit II (Miltenyi Biotec, USA) following Ficoll density gradient centrifugation, and then were seeded in 24-well plate (1×10^6^ cells/well) or 96-well plate (1.2×10^5^ cells/well) with the stimulation of ImmunoCult™ Human CD3/CD28 T Cell Activator (STEMCELL, Canada). Then CD4+ T cells were co-cultured with hUC-MSC-sEVs-treated macrophages at a ratio of 40:1 (CD4+ T cells: macrophages). After 5 days, CD4+ T cells were prepared for flow cytometric analysis or Bromodeoxyuridine (BrdU) assay.

### Bromodeoxyuridine (BrdU) Assay

Activated human CD4+ T cells treated with hUC-MSC-sEVs-educated macrophages were incubated with BrdU labelling reagent (10 μM) for the last 24h. Cell proliferation was quantitatively determined based on the measurement of BrdU incorporation, using a cell proliferation ELISA kit (Roche Diagnostics GmbH, Germany) following manufacturer’s instructions.

### Flow Cytometric Analysis

The lymphocytes were isolated from LGs as described previously (26). The lymphocytes in LGs, rabbit PBMCs treated by hUC-MSC-sEVs or human CD4+ T cell co-cultured with macrophages were fixed, permeabilized and stained with mouse anti-human Foxp3-PE (clone 206D), rat anti-human CD4-FITC (clone A161A1), mouse anti-human CD25-APC (clone BC96) (BioLegend, USA), and mouse anti-rabbit CD4-FITC (clone KEN-4, BIO-RAD, USA). Stained cells were analyzed with FACS Calibur flow cytometer (BD Biosciences, USA).

### Quantitative Real-Time PCR (Q-PCR)

Total RNA from LGs or cells was extracted using EZ-press RNA Purification Kit (EZBioscience, USA) following manufacturer’s instructions. Quantitative real-time PCR (Q-PCR) was performed using Sybr Green Master Mix and an ABI 7900 HT Sequence Detection System (both from Thermo Fisher Scientific, USA). The relative expression was calculated using the following equation: relative gene expression= 2^[ΔCt(control)–ΔCt(target)]^.

Total RNA of hUC-MSC-sEVs was isolated using Exosome RNA Purification Kit (EZBioscience, USA). Additionally, the synthetic miRNA Caenorhabditis elegans miR-39 (cel-miR-39, 200fmol/μl, Sequence: UCACCGGGUGUAAAUCAGCUUG), was added to the isolated total RNA, and was used as an exogenous control (27). The steps for real-time amplification were the same as above.

The sequences of the primers are listed in **Table S1**-**S3**.

### Immunofluorescence Staining

After dewaxed and rehydrated, LG tissue paraffin sections were subjected to citric acid antigen retrieval. Then sections were incubated with CD68 antibody (clone KP1, 1:50, Abcam, UK) overnight at 4°C after blocking with 1% goat serum, followed by incubating with anti-mouse IgG H&L (Alexa Fluor 488) secondary antibody (1: 500, Abcam, UK) at room temperature for 2 h away from the light. Nuclear DNA was labelled with DAPI. Images were observed with laser scanning confocal microscope (LSM800, Zeiss, Germany).

### Western Blot Analysis

Proteins isolated from cells, tissues or sEVs were loaded on 8-10% SDS-PAGE and transferred onto PVDF membrane. The membranes were blocked with 5% skimmed milk at RT for 1 h and further incubated with mouse antibodies against β-actin (clone OTI1, 1:2000, ZSGB-BIO, China), iNOS/NOS2 (clone C-11, 1:500, Santa Cruz, USA), Arg1 (clone C1, 1:500), CD9 (clone MEM-61, 1:500), CD63 (clone TS63, 1:500), CD81(clone M38, 1:500), TSG101 (clone 4A10, 1:500), or Calnexin (clone 6F12BE10, 1:500) (Abcam, UK) overnight at 4°C. Detection was carried out using anti-mouse IgG, HRP-linked secondary antibody (1:1000, CST, USA) for 2 h at room temperature. The bands were visualized using Tanon 4800 Multispectral Imaging System (Tanon Science & Technology, China).

### Statistical Analysis

Data from at least three independent experiments were presented as mean ± SD. The Shapiro-Wilk test was used to test the normality of the data. Comparisons between groups were made using the Student’s t test and one-way ANOVA, or Mann-Whitney U test and Kruskal-Wallis test according to their distribution. All analyses were performed using SPSS 25.0 software (IBM Corporation, USA), and a value of P ≤ 0.05 was considered statistically significant.

## Results

### Characterization of hUC-MSCs and hUC-MSC-sEVs

**Figure 1:**
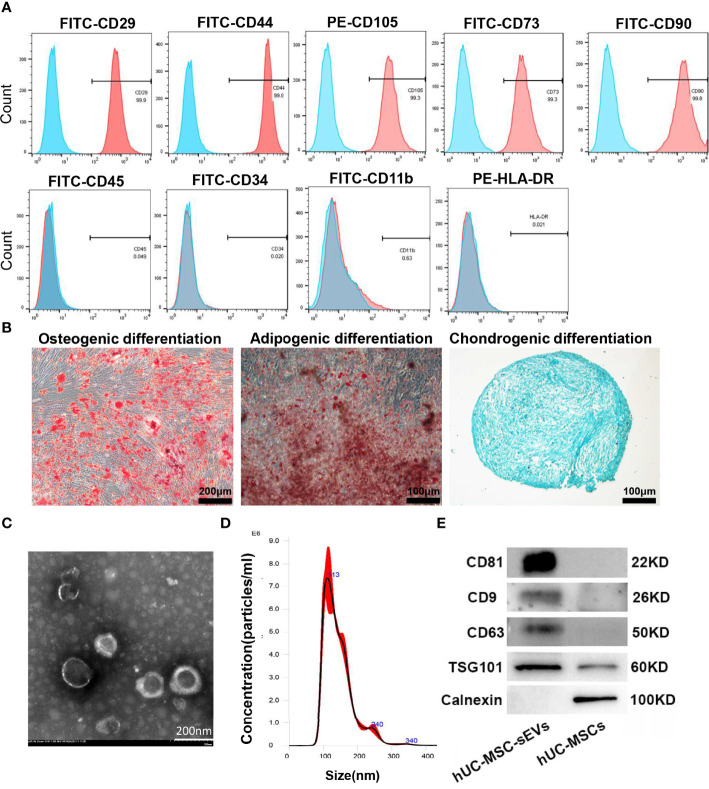
Characterization of hUC-MSCs and hUC-MSC-sEVs. **(A)** Flow cytometry analysis for the immunophenotypic surface markers (CD29, CD44, CD105, CD73, CD90, CD45, CD34, CD11b and HLA-DR) of hUC-MSCs. Blue histograms represent the isotype controls, and the red solid peak represents the marker indicated. **(B)** Representative images of osteogenic (alizarin red S staining), adipogenic (Oil Red O staining) and chondrogenic (Alcian Blue staining) differentiation assay. **(C)** Representative transmission electron micrograph of hUC-MSC-sEVs. **(D)** Nanoparticle tracking analysis for measurement of hUC-MSC-sEVs size. **(E)** Western blotting of marker proteins (CD81, CD9, CD63, TSG101 and Calnexin) in hUC-MSC-sEVs and hUC-MSCs.

The hUC-MSCs we obtained were positive for CD29, CD44, CD105, CD73, CD90 and negative for CD45, CD34, CD11b and MHC Class II antigen (HLA-DR) (**Figure 1A**), and were able to differentiate into adipocytes, osteocytes and chondrocytes under *in vitro* culture (**Figure 1B**). hUC-MSC-sEVs were purified from the conditioned medium of hUC-MSCs by serial differential centrifugation plus ultracentrifugation. Examination of TEM revealed that hUC-MSC-sEVs were round or ellipsoid, cup-shaped with double layer membrane structure (**Figure 1C**). NTA demonstrated that the diameters of these particles mainly ranged from 50-150 nm with a peak at around 113 nm (**Figure 1D**). Using western blot, we further confirmed that these nanovesicles were positive for specific markers: CD81, CD9, CD63 and TSG101, and were negative for the non-sEV marker Calnexin (**Figure 1E**).

### hUC-MSC-sEVs Administration Efficiently Prevented the Development of Rabbit Autoimmune Dacryoadenitis

**Figure 2:**
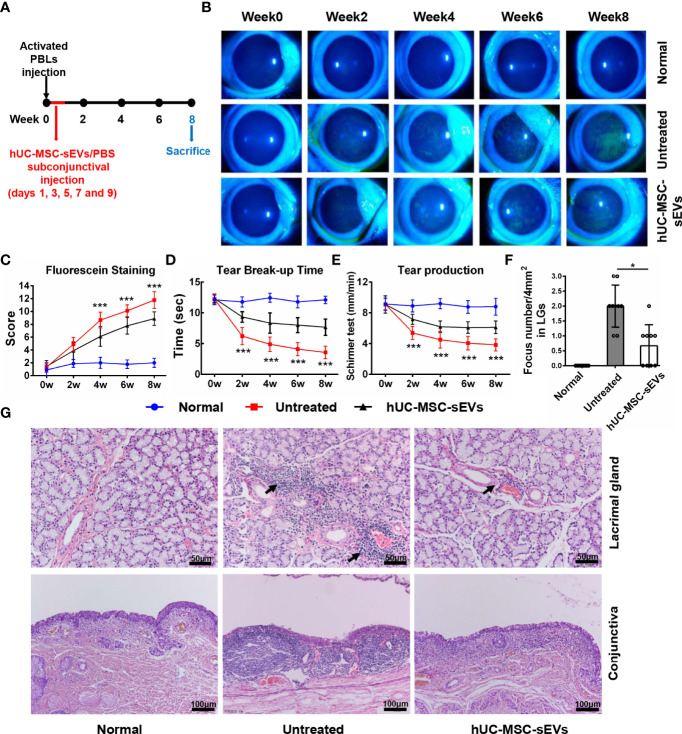
The preventive effects of hUC-MSC-sEVs on rabbit autoimmune dacryoadenitis. **(A)** Schematic diagram of hUC-MSC-sEVs administered at the early stage of rabbit autoimmune dacryoadenitis. **(B, C)** Representative images and scores of fluorescein staining in cornea. **(D)** The mean values of tear break-up time. **(E)** Quantification of tear production by Schirmer’s test. **(F)** Quantification of the numbers of lymphocytic foci per 4mm^2^ in LGs. **(G)** Representative photographs of H&E staining in LGs and conjunctivas. Arrows indicate infiltrating lymphocytes. n = 9 rabbits per group **(C–F)**. Data were shown as mean± SD. * hUC-MSC-sEVs group versus untreated group, *P < 0.05, **P < 0.01, ***P < 0.001.

The rabbit autoimmune dry eye model usually developed obvious clinical symptoms on the second week after adoptive transfer of activated PBLs (25). To investigate whether hUC-MSC-sEVs have preventive effects on autoimmune dacryoadenitis in rabbits, we subconjunctivally injected hUC-MSC-sEVs or PBS into rabbits during the early stage after adoptive transfer (days 1, 3, 5, 7 and 9) (**Figure 2A**), and observed the clinical signs until the eighth week. The hUC-MSC-sEVs administration attenuated the disease severity as early as 2 weeks after transfer. Compared with the untreated group, the hUC-MSC-sEVs group displayed milder corneal epithelial damage, more tear secretion and longer tear BUT at all the time points observed (**Figures 2B-E**). To further validate the effect of hUC-MSC-sEVs, the histological examination was performed on week 8 after transfer. As shown in **Figures 2F-G**, less infiltration of inflammatory cells and more minor tissue damage were observed in LGs and conjunctivas of rabbits in the hUC-MSC-sEVs group when compared to those without hUC-MSC-sEVs administration. All together, these findings suggested that hUC-MSC-sEVs administered in the early stage of disease were effective in attenuating the clinical severity and reducing lacrimal and conjunctival inflammation in induced rabbit dry eye model.

### hUC-MSC-sEVs Administration Promoted M2 Polarization and Induced Treg Generation *In Vivo*

**Figure 3:**
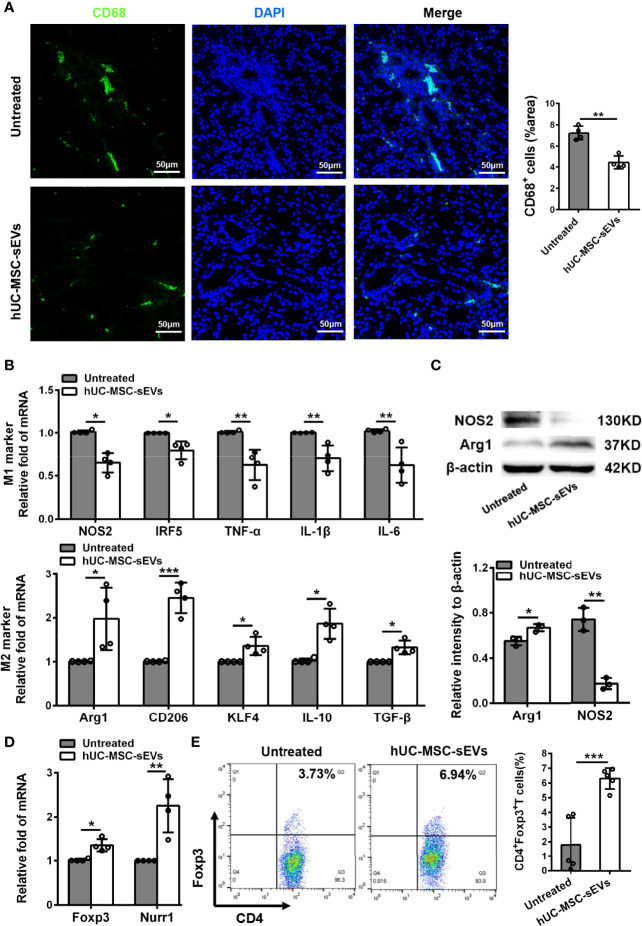
hUC-MSC-sEVs administration promoted M2 macrophage polarization and induced Treg generation *in vivo*. LGs were collected from rabbits in the untreated and hUC-MSC-sEVs group at week 8. **(A)** Representative immunofluorescent histological images of CD68 (pan macrophage marker) expression in LGs, and quantification of percent area of CD68+ cells. **(B)** Gene expression of M1 macrophage markers (NOS2, IRF5, TNF-α, IL-1β and IL-6) and M2 macrophage markers (Arg1, CD206, KLF4, IL-10 and TGF-β) in LGs. **(C)** Representative western blot images and quantification of relative band intensities for NOS2 and Arg1 in LGs. **(D)** Gene expression of Foxp3 and Nurr1 in LGs. **(E)** Flow cytometric analysis of the percentage of CD4+Foxp3+T cells among lymphocytes isolated from LGs. Representative data from at least three independent experiments were presented as mean± SD. *P < 0.05, **P < 0.01, ***P < 0.001.

Macrophages are important regulators in inflammation of LGs (5, 28). To clarify its underlying mechanism of how hUC-MSC-sEVs prevented the rabbit autoimmune dacryoadenitis, we investigated the effects of hUC-MSC-sEVs on macrophages accumulated in inflamed LGs. Immunofluorescence assay showed that the expression of CD68, a typical pan macrophage marker, was lower in perivenular/periductal area of LGs from model rabbits treated with hUC-MSC-sEVs than those from untreated groups (**Figure 3A**). It is well recognized that macrophages exhibit the ability to switch between M1 and M2 in response to microenvironmental changes (29). We therefore asked whether hUC-MSC-sEVs could regulate macrophage polarization. LGs collected from rabbits with or without hUC-MSC-sEVs administration were applied for Q-PCR and western blot. As shown in **Figure 3B**, in comparison with those of the untreated group, the gene expression of M1 markers nitric oxide synthase 2 (NOS2), interferon-regulatory factor 5 (IRF5), TNF-α, IL-1β and IL-6 was significantly reduced, whereas the gene expression of M2 markers, such as arginase-1 (Arg1), CD206, krueppel-like factor 4 (KLF4), IL-10 and TGF-β, was significantly increased in LGs of hUC-MSC-sEVs group. Consistently, the western blot analysis showed that hUC-MSC-sEVs administration led a dramatic increase in protein level of Arg-1 and a significantly reduced NOS2 protein expression in LGs (**Figure 3C**). Collectively, these findings suggested that hUC-MSC-sEVs could convert macrophages into the anti-inflammatory M2 phenotype in response to the chronic inflammation in LGs. These M2-like macrophages may have beneficial effects on alleviating autoimmune dacryoadenitis through secreting anti-inflammatory cytokines and promoting tissue repair.

Considering the critical role of Tregs in preventing lacrimal gland autoimmunity (14), we next examined the effect of hUC-MSC-sEVs on Tregs. Tregs comprise a distinct anti-inflammatory lineage specified by the X-linked transcription factor Foxp3, which can be directly regulated by the orphan nuclear receptor, Nurr1 (30). We observed that the mRNA expression of Foxp3 and Nurr1, and the proportion of CD4+Foxp3+Tregs in inflamed LGs were significantly elevated after hUC-MSC-sEVs administration (**Figures 3D, E**). Together, these data indicated that hUC-MSC-sEVs administration promoted Treg induction in rabbit autoimmune dacryoadenitis.

### hUC-MSC-sEVs Administered at the Developed Stage Have Therapeutic Effects on Rabbit Autoimmune Dacryoadenitis

**Figure 4:**
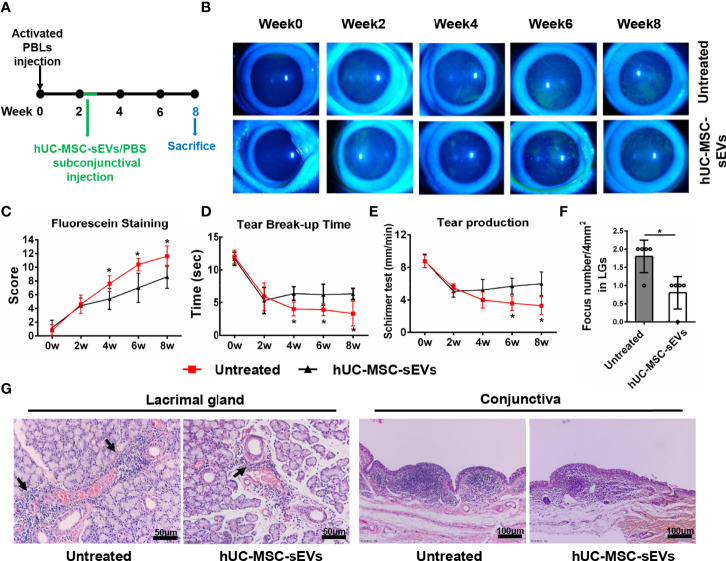
The therapeutic effects of hUC-MSC-sEVs on rabbit autoimmune dacryoadenitis. **(A)** Schematic representation of hUC-MSC-sEVs administered at the developed stage of rabbit autoimmune dacryoadenitis. **(B, C)** Corneal fluorescein staining images and grading scores. **(D)** Tear break-up time. **(E)** Tear production. **(F)** Numbers of lymphocytic foci per 4 mm^2^ in LGs. **(G)** Representative specimens of H&E staining in LGs and conjunctivas. Arrows indicate infiltrating lymphocytes. n = 5 rabbits per group **(C–F)**. Data were shown as mean± SD. *P < 0.05.

In order to observe the therapeutic effects of hUC-MSC-sEVs *in vivo*, we started treating model rabbits with every-two-day injection of hUC-MSC-sEVs or PBS at the developed stage of the disease (2 weeks after transfer) for 5 doses (**Figure 4A**). As shown in **Figures 4B-E**, the relief of clinical signs was observed 2 weeks after first hUC-MSC-sEVs injection. Treatment with hUC-MSC-sEVs resulted in significantly lower fluorescein staining scores and obviously increased BUT from week 4 to week 8, and remarkably increased aqueous tear volume at week 6 and 8 in rabbits compared with those in the untreated group. Histologically, immune cell infiltrates were diminished in LGs and conjunctivas of model rabbits by hUC-MSC-sEVs administration (**Figures 4F, G**). As expected, these results indicated that hUC-MSC-sEVs administered at the developed stage of diseases alleviated the severity of rabbit autoimmune dacryoadenitis.

### hUC-MSC-sEVs Regulated M2 Macrophage Polarization and Treg Cell Differentiation *In Vitro*

**Figure 5:**
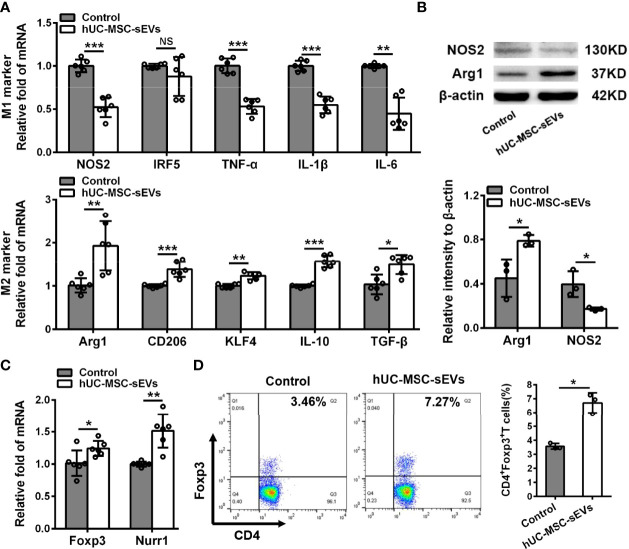
hUC-MSC-sEVs regulated M2 macrophage polarization and Treg differentiation *in vitro*. PBMCs isolated from model rabbits were stimulated with irradiated pLGECs, and then were treated with or without hUC-MSC-sEVs (5 μg/ml). **(A)** Gene expression profiles of M1 markers (NOS2, IRF5, TNF-α, IL-1β and IL-6) and M2 markers (Arg1, CD206, KLF4, IL-10 and TGF-β) in PBMCs. **(B)** Western blot analysis of NOS2 and Arg1 protein level in PBMCs. **(C)** The mRNA expression of Foxp3 and Nurr1 in PBMCs. **(D)** Representative flow cytometry showing the frequency of CD4+Foxp3+ T cells in PBMCs. Data were from at least three independent experiments and presented as mean± SD. *P < 0.05, **P < 0.01, ***P < 0.001, NS, not significant.

We next investigated the modulatory effects of hUC-MSC-sEVs on M2 macrophage polarization and Treg cell differentiation *in vitro*. PBMCs from model rabbits stimulated with irradiated pLGECs were treated with or without hUC-MSC-sEVs (5 μg/ml). Firstly, we observed that the transcript levels of M1 markers (NOS2, TNF-α, IL-1β and IL-6) were obviously reduced, whereas the mRNA levels of M2 markers (Arg1, CD206, KLF4, IL-10 and TGF-β) were significantly increased in the hUC-MSC-sEVs group compared with the control group, which was consistent with the protein expression of NOS2 and Arg1 (**Figures 5A, B**). Then we evaluated the effect of hUC-MSC-sEVs on Tregs. The Q-PCR analysis revealed the significantly higher expression of Foxp3 and Nurr1 in the hUC-MSC-sEVs group than that of the control group (**Figure 5C**). Flow cytometry analysis of PBMCs collected after 5-day co-culture showed that hUC-MSC-sEVs treatment significantly increased the percentage of CD4+Foxp3+Tregs (**Figure 5D**). Taken together, these data demonstrated that hUC-MSC-sEVs could induce M2 polarization and promote Treg generation in stimulated PBMCs.

### hUC-MSC-sEVs Converted Inflammatory Macrophages to the M2 Phenotype, Which Suppressed T Cell Proliferation and Increased Tregs

**Figure 6:**
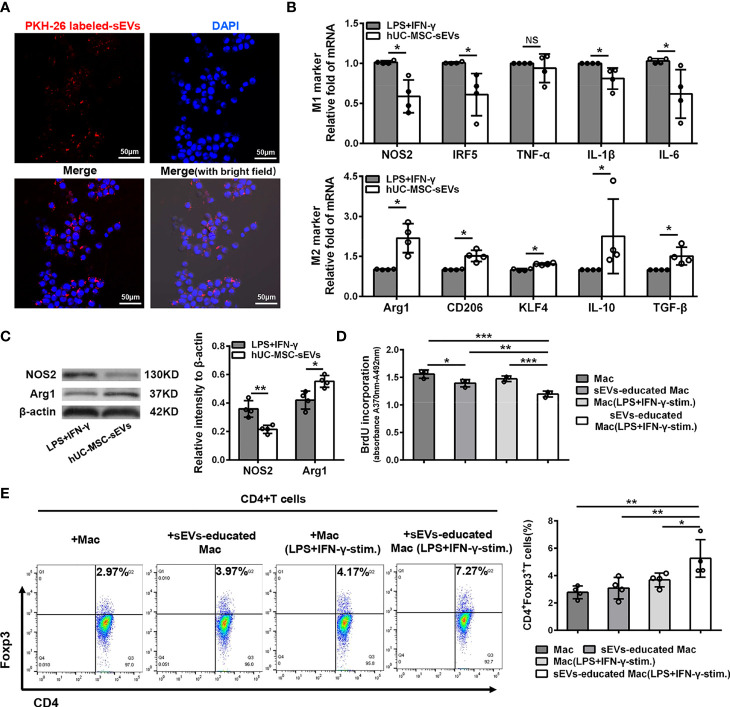
hUC-MSC-sEVs converted inflammatory macrophages to M2 phenotype, which suppressed T cell proliferation and increased Treg frequency. **(A)** Representative images of the uptake of PKH-26-labeled hUC-MSC-sEVs (red) by human-monocyte-differentiated macrophages (called as Mac, DAPI blue). **(B, C)** LPS+IFN-γ-stimulated Mac was co-cultured with or without 5 μg/ml hUC-MSC-sEVs for 48h. **(B)** Q-PCR analysis of M1 markers (NOS2, IRF5, TNF-α, IL-1β and IL-6) and M2 markers (Arg1, CD206, KLF4, IL-10 and TGF-β). **(C)** Western blot assay for NOS2 and Arg1 protein level. **(D, E)** After incubated with hUC-MSC-sEVs, macrophages were collected and cultured together with human CD4+ T cells for 5 days in the presence of anti-CD3/-CD28 antibodies. **(D)** BrdU assay for CD4+ T cell proliferation. **(E)** Flow cytometric analysis for CD4+Foxp3+ T cells. sEVs, hUC-MSC-sEVs; LPS+IFN-γ-stim., LPS+ IFN-γ-stimulation. Representative data from at least three independent experiments were presented as mean± SD. *P < 0.05, **P < 0.01, ***P < 0.001, NS, not significant.

Given the essential role of macrophages in innate and adaptive immunity, we next focus on the influence of hUC-MSC-sEVs on macrophages. Under confocal laser-scanning microscope, we observed that the PKH26 (red)-labeled hUC-MSC-sEVs were internalized into PMA-treated THP-1 cells (called as Mac) after six-hour co-incubation and localized in the cytoplasm (**Figure 6A**). To further validate the role of hUC-MSC-sEVs on macrophage phenotype, LPS+IFN-γ-stimulated Mac was co-cultured with or without hUC-MSC-sEVs for 48h. The levels of M1 and M2 markers were detected by Q-PCR and western blot analysis. The results showed that hUC-MSC-sEVs could effectively elicit human M1 macrophage to an anti-inflammatory M2 state *in vitro* (**Figures 6B, C**).

Previous studies have shown that MSC-sEVs required other mediator cells such as monocytes to promote Treg expansion (31, 32), and anti-inflammatory macrophages could induce Tregs (33). Hence, we sought to examine whether the macrophages educated by hUC-MSC-sEVs influenced T cell proliferation and Treg differentiation. After incubated with hUC-MSC-sEVs as aforementioned, Mac and LPS+IFN-γ -stimulated Mac were isolated from each group and co-cultured with human peripheral blood-derived CD4+ T cells in the presence of anti-CD3/-CD28 antibodies for 5 days. The BrdU proliferation assay revealed that CD4+ T cell proliferation was significantly repressed by hUC-MSC-sEVs-educated macrophages, especially those pre-stimulated with LPS+IFN-γ (**Figure 6D**). In addition, the flow cytometric analysis showed that hUC-MSC-sEVs-educated macrophages (with LPS+IFN-γ pre-stimulation) significantly elevated the proportion of CD4+Foxp3+Tregs compared with other groups (**Figure 6E**). These combined findings showed that hUC-MSC-sEVs-educated macrophages with M2-like phenotype could suppress CD4+ T proliferation and augment Treg generation.

### miR-100-5p Transferred by hUC-MSC-sEVs Influenced Macrophage Phenotype and Function

**Figure 7:**
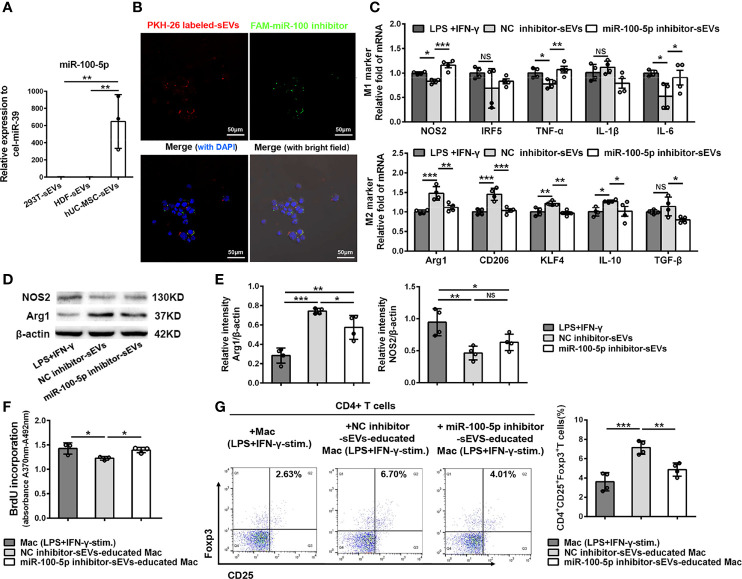
miR-100-5p transferred by hUC-MSC-sEVs modulated macrophage phenotype and function. **(A)** Q-PCR analysis of miR-100-5p levels in hUC-MSC-sEVs, 293T-sEVs and HDF-sEVs. **(B)** Representative images of the internalization of miR-100 inhibitor transfected (green) PKH-26-labeled hUC-MSC-sEVs (red) by THP-1 derived macrophages (DAPI blue). **(C–E)** LPS+IFN-γ-stimulated Mac was co-cultured with miR-100-5p inhibitor-sEVs or NC inhibitor-sEVs for 48h. **(C)** Q-PCR analysis of M1 markers (NOS2, IRF5, TNF-α, IL-1β and IL-6) and M2 markers (Arg1, CD206, KLF4, IL-10 and TGF-β). **(D, E)** Protein analysis for NOS2 and Arg1. **(F, G)** THP-1 derived macrophages pretreated with miR-100-5p inhibitor-sEVs or NC inhibitor-sEVs were co-cultured with human CD4+ T cells stimulated by anti-CD3/-CD28. **(F)** BrdU assay for CD4+ T cell proliferation. **(G)** Flow cytometry for CD4+CD25+Foxp3+ T cells. sEVs, hUC-MSC-sEVs; LPS+IFN-γ-stim., LPS+IFN-γ-stimulation. NC, negative-control. Representative data from at least three independent experiments were presented as mean± SD. *P < 0.05, **P < 0.01, ***P < 0.001, NS, not significant.

Increasing evidence have shown that miRNAs are the important functional cargos in sEVs (34, 35). Therefore, we determined to investigate whether hUC-MSC-sEVs contained any pivotal miRNAs that contributed to their immunomodulatory effects on macrophages. We analyzed the miRNA sequencing outcomes of hUC-MSC-sEVs reported in three published studies (36–38), and aligned them to a public available database of exosomal miRNA profiles (ExoCarta). The top 5 miRNAs from each of the sequencing data were shown in **Table S4**. Among them, miR-100-5p attracted our attention: First, it was one of most highly enriched miRNAs in hUC-MSC-sEVs in comparison with both sEVs derived from human embryonic kidney 293T cells (293T-sEVs) and human dermal fibroblasts (HDF-sEVs). Second, it was previously reported to be crucial in maintaining tumor-associated macrophage M2 phenotype (39). Thus, we hypothesized that sEVs-miR-100-5p might play a role in sEVs-mediated M2 polarization. To test this, we performed Q-PCR assay and confirmed that miR-100-5p was significantly highly expressed in hUC-MSC-sEVs we isolated (**Figure 7A**). In addition, we found that miR-100-5p in LGs of model rabbits was significantly upregulated after treatment with hUC-MSC-sEVs (**Figure S1**), suggesting that miR-100-5p may be helpful for improved rabbit autoimmune dacryoadenitis. Subsequently, in order to explore the direct effects of miR-100-5p on macrophage polarization *in vitro*, LPS+IFN-γ-stimulated Mac were transfected with miR-100-5p mimics or NC mimics. The results showed that miR-100-5p overexpression significantly facilitated the polarization of macrophages from M1 to M2 phenotype (**Figure S2**). Collectively, these data indicated that miR-100-5p might be the key components contributing to the regulation of hUC-MSC-sEVs on M2 macrophage polarization.

To further confirm the role of miR-100-5p in hUC-MSC-sEVs, sEVs purified from the conditioned medium of hUC-MSCs transfected with miR-100-5p inhibitor, NC inhibitor, miR-100-5p mimics or NC mimics (called as miR-100-5p inhibitor-sEVs, NC inhibitor-sEVs, miR-100-5p mimics-sEVs or NC mimics-sEVs, respectively), were co-cultured with macrophages as mentioned before. The transfection efficiency was confirmed by Q-PCR analysis (**Figure S3**). We observed that the FAM (green)-labeled miR-100-5p inhibitor was contained in PKH26 (red)-labeled sEVs, which were internalized into macrophages after 6 hours of co-incubation (**Figure 7B**). Further analysis revealed that, compared with NC inhibitor-sEVs, miR-100-5p inhibitor-sEVs were less effective in suppressing M1 marker expression and enhancing M2 marker expression (**Figures 7C-E**), indicating that inhibiting miR-100-5p in hUC-MSC-sEVs blunted their effects on M2 macrophage polarization. Furthermore, miR-100-5p mimics-sEVs markedly upregulated M2 markers and downregulated M1 markers compared to those NC mimics-sEVs (**Figures S4A, B**). These results together support the notion that miR-100-5p was involved in the hUC-MSC-sEVs mediated M2 macrophage polarization.

Next, we explored whether miR-100-5p in hUC-MSC-sEVs influenced the function of hUC-MSC-sEVs-educated macrophages on T cells. To this end, THP-1 derived macrophages pretreated with miR-100-5p inhibitor-sEVs or NC inhibitor-sEVs were collected and then co-cultured with human CD4+ T cells. The BrdU proliferation assay showed that CD4+ T cell proliferation was markedly inhibited by NC inhibitor-sEVs-educated macrophages, not by miR-100-5p inhibitor-sEVs-educated macrophages (**Figure 7F**). Likewise, the percentage of the CD25+Foxp3+Treg populations was significantly lower in CD4+ T cells co-cultured with miR-100-5p inhibitor-sEVs-educated macrophages than in those co-cultured with NC inhibitor-sEVs-educated macrophages (**Figure 7G**), whereas the percentage of the CD4+Foxp3+Treg populations was obviously upregulated by miR-100-5p mimics-sEVs-educated macrophages (**Figure S4C**). Hence, these findings indicated that miR-100-5p in hUC-MSC-sEVs was, at least, partly responsible for the T cell suppression and Treg cell expansion induced by hUC-MSC-sEVs-educated macrophages.

## Discussion

In the present study, we firstly demonstrated the preventive and therapeutic effects of hUC-MSC-sEVs on autoimmune dacryoadenitis in rabbits. We further confirmed that hUC-MSC-sEVs administration promoted the polarization of M2 macrophages and the generation of Tregs, which might suppress inflammation and enhance subsequent tissue reparative activities, thereby alleviate the disease severity. Furthermore, we demonstrated that after treatment with hUC-MSC-sEVs, macrophages were favorably shifted to an anti-inflammatory M2 phenotype, which could inhibit the proliferation of CD4+ T cells and increase the frequency of Tregs. Importantly, we discovered that miR-100-5p abundant in hUC-MSC-sEVs participated in the regulation of hUC-MSC-sEVs on M2 macrophage polarization and the effect of hUC-MSC-sEVs-educated macrophages on Tregs.

Being as effective immunomodulators as their parent cells, MSC-sEVs have been increasingly proved as promising therapeutic alternatives against some autoimmune and inflammatory disorders due to their *in vivo* stability, hypo-immunogenicity and low risk of tumorigenicity (18, 40). For example, Bai et al. (41) demonstrated that periocularly injected MSC-sEVs on the onset of the disease could effectively ameliorate experimental autoimmune uveitis. Limited data, however, is available regarding the possible effect of MSC-sEVs on SS dry eye. In a NOD mouse model of SS, Hai et al. (42) reported that sEVs from human induced pluripotent stem cell (iPSC)-MSCs infused at the pre-disease stage have the potential to alleviate sialadenitis, but they didn’t mention the effect on ocular manifestations. Although Kim et al. (43) have proved that single injection of bone marrow MSC derived EVs into the intraorbital LGs could improve inflammatory dry eye in mouse SS model during the one-week observation, they didn’t follow up its long-term therapeutic efficacy and didn’t explore its modulatory effects on macrophages. In our study, we found that hUC-MSC-sEVs were effective in controlling rabbit autoimmune dacryoadenitis when administered subconjunctivally during the early stage or the developed stage of the disease. Long-lasting efficacy was demonstrated by improved clinical scores and diminished tissue impairment of LGs and conjunctivas during an observation period of 42 or 56 days.

M1/M2 macrophages play a key role in the onset or development of autoimmune lesions in SS (44). Increased M1 macrophage polarization and decreased M2 macrophage polarization in LGs might contribute to the pathogenesis of autoimmune dacryoadenitis (6). In this study, we demonstrated that hUC-MSC-sEVs treatment decreased M1 activation and skewed macrophages to the M2 phenotype in rabbit SS dry eye model, which was consistent with previous studies in other animal models (45, 46). KLF4 is the key transcription factors involved in macrophage polarization, and is found to cooperate with STAT6 to augment M2 gene expression and inhibit M1 gene transcription *via* inhibiting NF-κB activation (47). Here, we found that hUC-MSC-sEVs could significantly enhance the transcription of KLF4 both *in vivo* and *in vitro*, suggesting that KLF4 was an important driver for hUC-MSC-sEVs-induced M2 polarization. These M2-like macrophages were prone to release anti-inflammatory mediators, such as Arg1, IL-10 and TGF-β, to downregulate the M1 macrophage responses (48) and even influence the activity of other immune cells, subsequently contributing to dampening excessive inflammation and preventing LG dysfunction.

Tregs, which specifically expressed the forkhead transcription factor Foxp3, are known to attenuate immune activity (49), and the beneficial effects of MSC-sEVs-mediated enhanced generation of Tregs have been well documented in several autoimmune disorders (50, 51). In agree with these, we found that hUC-MSC-sEVs dramatically enhanced the proportion of CD4+Foxp3+Tregs both *in vivo* and *in vitro*. Nurr1 (NR4A2), one member of orphan nuclear receptor NR4A family, has been shown to bind to regulatory regions of Foxp3, and regulate the differentiation and function of Tregs by promoting Foxp3 expression and repressing inflammatory cytokine production (52, 53). Our data firstly showed that hUC-MSC-sEVs enhanced the expression of Nurr1, indicating that hUC-MSC-sEVs might promote CD4+Foxp3+Treg function *via* regulation of Nurr1 activity, but the underlying mechanism still needs further investigation. On the contrary, a study by Cosenza et al. (16) reported that MSC-sEVs did not influence the percentage of Tregs in collagen-induced arthritis model. Different cellular source of sEVs and disease context may explain the discrepancy among these studies.

Both M2 macrophages and Tregs have key roles in maintaining immune tolerance, and have been recently shown to cooperate with each other to enhance immunosuppression in tumors (54). Our data demonstrated that the increased M2 marker expression was positively correlated with the elevated Treg percentage in hUC-MSC-sEVs-treated dry eye model, suggesting these two types of cells may interact with each other in hUC-MSC-sEVs-induced immune-modulatory milieu. Indeed, our *in vitro* results revealed that hUC-MSC-sEVs-educated macrophages with the most typical M2 features had strong capacity to increase the percentage of Tregs and suppress CD4+ T cell proliferation. In turn, Tregs are able to induce the generation of M2 macrophages (55), thereby forming a loop for further amplification of Treg production. This positive-feedback loop between M2 macrophages and Tregs by promoting generation of each cell type may significantly contribute to the suppressive effects of hUC-MSC-sEVs on autoimmune dacryoadenitis.

miRNAs are widely accepted as important components of MSC-sEVs and largely decide the modulatory effects of MSC-sEVs on recipient cells (56–58). miR-100-5p is a highly conserved non-coding RNA with anti-inflammatory function (59). It has been reported that miR-100-5p could inhibit IL-6 and TLR4 mRNA gene expression in follicular dendritic cells during germinal center reactions (60), suppress inflammatory activation of microglia (61) and promote tumor-associated macrophage M2 phenotype (39). As one of highly enriched miRNAs in hUC-MSC-sEVs (36–38, 62), miR-100-5p delivered by MSC-sEVs was found to protect cartilage from damage in osteoarthritis, and inhibit NLRP3 inflammasome activation and suppress cytokine release to ameliorate myocardial ischemia/reperfusion injury (63, 64). Howbeit, the effect of sEVs-derived miR-100-5p on macrophage polarization remains uninvestigated so far. In this study, we firstly demonstrated that miR-100-5p overexpressed hUC-MSC-sEVs promoted M2 macrophage polarization, whereas blocking miR-100-5p in hUC-MSC-sEVs blunted the regulation of hUC-MSC-sEVs on M2 macrophage polarization, and even weakened the effect of hUC-MSC-sEVs-educated macrophages on T cell suppression and Treg cell expansion, suggesting that the sEVs-mediated transfer of miR-100-5p is an important mechanism for the immunomodulatory effects of hUC-MSC-sEVs on macrophage phenotype and function. Additionally, the expression of miR-100-5p in rabbit autoimmune dacryoadenitis was significantly increased by hUC-MSC-sEVs administration. Recent study has reported that miR-100-5p could attenuate chronic vascular inflammation *via* repression of mammalian target of rapamycin (mTOR) signaling (63). Increased mTOR activation has been proved to play a crucial role in SS pathogenesis (65). Therefore, we supposed that through transferring miR-100-5p, hUC-MSC-sEVs may inhibit mTOR pathway to polarize macrophage to the M2 phenotype, leading to alleviated autoimmune dacryoadenitis, and further investigation is needed.

## Conclusion

In summary, our study demonstrated that hUC-MSC-sEVs administration attenuated rabbit autoimmune dacryoadenitis by skewing macrophages into a M2 phenotype and increasing Treg proportion. It’s worth noting that the sEVs-miR-100-5p had a critical role in the immunomodulatory function of hUC-MSC-sEVs, particularly on macrophages, which ultimately possessed T-cell immunosuppressive properties. These results suggest that management of hUC-MSC-sEVs and their functional cargoes may provide a potential therapeutic strategy for the prevention and treatment of autoimmune dry eye.

## Data Availability Statement

The raw data supporting the conclusions of this article will be made available by the authors, without undue reservation.

## Ethics Statement

The studies involving human participants were reviewed and approved by the Ethics Committee of Tianjin Medical University Eye Hospital. The ethics committee waived the requirement of written informed consent for participation. The animal study was reviewed and approved by the Laboratory Animal Care and Use Committee of Tianjin Medical University Eye Hospital.

## Author Contributions

NL: collection and assembly of data, data analysis and interpretation, manuscript writing. ZG and LZ: collection of data. BD and BM: Provision of study material. HN: conception and design, data analysis and interpretation, manuscript writing and final approval. RW: conception and design, financial support, manuscript writing and final approval. All authors contributed to the article and approved the submitted version.

## Funding

National Natural Science Foundation of China, Grant/Award Numbers: 82070929, 81970793, 81770901; Tianjin Clinical Key Discipline Project, Grant/Award Number: TJLCZDXKT003.

## Conflict of Interest

The authors declare that the research was conducted in the absence of any commercial or financial relationships that could be construed as a potential conflict of interest.

## Publisher’s Note

All claims expressed in this article are solely those of the authors and do not necessarily represent those of their affiliated organizations, or those of the publisher, the editors and the reviewers. Any product that may be evaluated in this article, or claim that may be made by its manufacturer, is not guaranteed or endorsed by the publisher.

## References

[1] Akpek EK Bunya VY Saldanha IJ. Sjogren's Syndrome: More Than Just Dry Eye. Cornea (2019) 38(5):658–61. doi: 10.1097/ICO.0000000000001865 PMC6482458 30681523

[2] Gliozzi M Greenwell-Wild T Jin W Moutsopoulos NM Kapsogeorgou E Moutsopoulos HM. A Link Between Interferon and Augmented Plasmin Generation in Exocrine Gland Damage in Sjogren's Syndrome. J Autoimmun (2013) 40:122–33. doi: 10.1016/j.jaut.2012.09.003 PMC4259572 23110742

[3] Park YS Gauna AE Cha S. Mouse Models of Primary Sjogren's Syndrome. Curr Pharm Des (2015) 21(18):2350–64. doi: 10.2174/1381612821666150316120024 PMC4425610 25777752

[4] Zhou D McNamara NA. Macrophages: Important Players in Primary Sjogren's Syndrome? Expert Rev Clin Immunol (2014) 10(4):513–20. doi: 10.1586/1744666x.2014.900441 24646086

[5] Zhou D Chen YT Chen F Gallup M Vijmasi T Bahrami AF. Critical Involvement of Macrophage Infiltration in the Development of Sjogren's Syndrome-Associated Dry Eye. Am J Pathol (2012) 181(3):753–60. doi: 10.1016/j.ajpath.2012.05.014 PMC3432423 22770665

[6] Lu X Li N Zhao L Guo D Yi H Yang L. Human Umbilical Cord Mesenchymal Stem Cells Alleviate Ongoing Autoimmune Dacryoadenitis in Rabbits *via* Polarizing Macrophages Into an Anti-Inflammatory Phenotype. Exp eye Res (2019) 191:107905. doi: 10.1016/j.exer.2019.107905 31891674 PMC8612174

[7] Funes SC Rios M Escobar-Vera J Kalergis AM. Implications of Macrophage Polarization in Autoimmunity. Immunology (2018) 154(2):186–95. doi: 10.1111/imm.12910 PMC5980179 29455468

[8] Sindrilaru A Peters T Wieschalka S Baican C Baican A Peter H. An Unrestrained Proinflammatory M1 Macrophage Population Induced by Iron Impairs Wound Healing in Humans and Mice. J Clin Invest (2011) 121(3):985–97. doi: 10.1172/JCI44490 PMC3049372 21317534

[9] Lee HS Amouzegar A Dana R. Kinetics of Corneal Antigen Presenting Cells in Experimental Dry Eye Disease. BMJ Open Ophthalmol (2017) 1(1):e000078. doi: 10.1136/bmjophth-2017-000078 PMC5721641 29354712

[10] You IC Coursey TG Bian F Barbosa FL de Paiva CS Pflugfelder SC. Macrophage Phenotype in the Ocular Surface of Experimental Murine Dry Eye Disease. Arch Immunol Ther Exp (Warsz) (2015) 63(4):299–304. doi: 10.1007/s00005-015-0335-0 25772203 PMC4523394

[11] Shapouri-Moghaddam A Mohammadian S Vazini H Taghadosi M Esmaeili SA Mardani F. Macrophage Plasticity, Polarization, and Function in Health and Disease. J Cell Physiol (2018) 233(9):6425–40. doi: 10.1002/jcp.26429 PMC13482560 29319160

[12] Aota K Yamanoi T Kani K Nakashiro KI Ishimaru N Azuma M. Inverse Correlation Between the Number of CXCR3(+) Macrophages and the Severity of Inflammatory Lesions in Sjogren's Syndrome Salivary Glands: A Pilot Study. J Oral Pathol Med (2018) 47(7):710–8. doi: 10.1111/jop.12756 29926992

[13] Di Benedetto P Ruscitti P Vadasz Z Toubi E Giacomelli R. Macrophages With Regulatory Functions, a Possible New Therapeutic Perspective in Autoimmune Diseases. Autoimmun Rev (2019) 18(10):102369. doi: 10.1016/j.autrev.2019.102369 31404701

[14] Lieberman SM Kreiger PA Koretzky GA. Reversible Lacrimal Gland-Protective Regulatory T-Cell Dysfunction Underlies Male-Specific Autoimmune Dacryoadenitis in the Non-Obese Diabetic Mouse Model of Sjogren Syndrome. Immunology (2015) 145(2):232–41. doi: 10.1111/imm.12439 PMC4427388 25581706

[15] Barr JY Wang X Kreiger PA Lieberman SM. Salivary-Gland-Protective Regulatory T-Cell Dysfunction Underlies Female-Specific Sialadenitis in the Non-Obese Diabetic Mouse Model of Sjogren Syndrome. Immunology (2018) 155(2):225–37. doi: 10.1111/imm.12948 PMC6142283 29750331

[16] Cosenza S Toupet K Maumus M Luz-Crawford P Blanc-Brude O Jorgensen C. Mesenchymal Stem Cells-Derived Exosomes Are More Immunosuppressive Than Microparticles in Inflammatory Arthritis. Theranostics (2018) 8(5):1399–410. doi: 10.7150/thno.21072 PMC5835945 29507629

[17] Yin K Wang S Zhao RC. Exosomes From Mesenchymal Stem/Stromal Cells: A New Therapeutic Paradigm. Biomark Res (2019) 7:8. doi: 10.1186/s40364-019-0159-x 30992990 PMC6450000

[18] Liu H Li R Liu T Yang L Yin G Xie Q. Immunomodulatory Effects of Mesenchymal Stem Cells and Mesenchymal Stem Cell-Derived Extracellular Vesicles in Rheumatoid Arthritis. Front Immunol (2020) 11:1912. doi: 10.3389/fimmu.2020.01912 32973792 PMC7468450

[19] Harrell CR Jovicic N Djonov V Arsenijevic N Volarevic V. Mesenchymal Stem Cell-Derived Exosomes and Other Extracellular Vesicles as New Remedies in the Therapy of Inflammatory Diseases. Cells (2019) 8(12):1605. doi: 10.3390/cells8121605 PMC6952783 31835680

[20] Cai J Wu J Wang J Li Y Hu X Luo S. Extracellular Vesicles Derived From Different Sources of Mesenchymal Stem Cells: Therapeutic Effects and Translational Potential. Cell Biosci (2020) 10:69. doi: 10.1186/s13578-020-00427-x 32483483 PMC7245623

[21] Qian X An N Ren Y Yang C Zhang X Li L. Immunosuppressive Effects of Mesenchymal Stem Cells-Derived Exosomes. Stem Cell Rev Rep (2020) 17(2):411–27. doi: 10.1007/s12015-020-10040-7 32935222

[22] Song Y Dou H Li X Zhao X Li Y Liu D. Exosomal miR-146a Contributes to the Enhanced Therapeutic Efficacy of Interleukin-1beta-Primed Mesenchymal Stem Cells Against Sepsis. Stem Cells (2017) 35(5):1208–21. doi: 10.1002/stem.2564 28090688

[23] Zhang H Zhang B Tao Y Cheng M Hu J Xu M. Isolation and Characterization of Mesenchymal Stem Cells From Whole Human Umbilical Cord Applying a Single Enzyme Approach. Cell Biochem Funct (2012) 30(8):643–9. doi: 10.1002/cbf.2843 22777760

[24] Sun J Sun X Chen J Liao X He Y Wang J. microRNA-27b Shuttled by Mesenchymal Stem Cell-Derived Exosomes Prevents Sepsis by Targeting JMJD3 and Downregulating NF-kappaB Signaling Pathway. Stem Cell Res Ther (2021) 12(1):14. doi: 10.1186/s13287-020-02068-w 33413595 PMC7791667

[25] Wei RH Thomas PB Samant DM Schechter JE Mircheff AK Trousdale MD. Autoimmune Dacryoadenitis and Sialadenitis Induced in Rabbits by Intravenous Injection of Autologous Lymphocytes Activated *Ex Vivo* Against Lacrimal Antigens. Cornea (2012) 31(6):693–701. doi: 10.1097/ICO.0b013e31823f8e47 22333667

[26] Li X Lu X Sun D Wang X Yang L Zhao S. Adipose-Derived Mesenchymal Stem Cells Reduce Lymphocytic Infiltration in a Rabbit Model of Induced Autoimmune Dacryoadenitis. Invest Ophthalmol Visual Sci (2016) 57(13):5161–70. doi: 10.1167/iovs.15-17824 PMC6016434 27699412

[27] Lovett JAC Durcan PJ Myburgh KH. Investigation of Circulating Extracellular Vesicle MicroRNA Following Two Consecutive Bouts of Muscle-Damaging Exercise. Front Physiol (2018) 9:1149. doi: 10.3389/fphys.2018.01149 30177888 PMC6109634

[28] Lee H Kim DH Hwangbo H Kim SY Ji SY Kim MY. The Protective Effect of Topical Spermidine on Dry Eye Disease With Retinal Damage Induced by Diesel Particulate Matter2. 5 Pharmaceutics (2021) 13(9):1439. doi: 10.3390/pharmaceutics13091439 34575516 PMC8468149

[29] Sica A Mantovani A. Macrophage Plasticity and Polarization: *In Vivo* Veritas. J Clin Invest (2012) 122(3):787–95. doi: 10.1172/jci59643 PMC3287223 22378047

[30] Park TY Jang Y Kim W Shin J Toh HT Kim CH. Chloroquine Modulates Inflammatory Autoimmune Responses Through Nurr1 in Autoimmune Diseases. Sci Rep (2019) 9(1):15559. doi: 10.1038/s41598-019-52085-w 31664129 PMC6820774

[31] Zhang B Yeo RWY Lai RC Sim EWK Chin KC Lim SK. Mesenchymal Stromal Cell Exosome-Enhanced Regulatory T-Cell Production Through an Antigen-Presenting Cell-Mediated Pathway. Cytotherapy (2018) 20(5):687–96. doi: 10.1016/j.jcyt.2018.02.372 29622483

[32] Du YM Zhuansun YX Chen R Lin L Lin Y Li JG. Mesenchymal Stem Cell Exosomes Promote Immunosuppression of Regulatory T Cells in Asthma. Exp Cell Res (2018) 363(1):114–20. doi: 10.1016/j.yexcr.2017.12.021 29277503

[33] Schmidt A Zhang XM Joshi RN Iqbal S Wahlund C Gabrielsson S. Human Macrophages Induce CD4(+)Foxp3(+) Regulatory T Cells *via* Binding and Re-Release of TGF-Beta. Immunol Cell Biol (2016) 94(8):747–62. doi: 10.1038/icb.2016.34 27075967

[34] Asgarpour K Shojaei Z Amiri F Ai J Mahjoubin-Tehran M Ghasemi F. Exosomal microRNAs Derived From Mesenchymal Stem Cells: Cell-to-Cell Messages. Cell Communication Signaling CCS (2020) 18(1):149. doi: 10.1186/s12964-020-00650-6 32917227 PMC7488404

[35] Zhang Y Kim M Jia B Yan J Zuniga-Hertz J Han C. Hypothalamic Stem Cells Control Ageing Speed Partly Through Exosomal miRNAs. Nature (2017) 548(7665):52–7. doi: 10.1038/nature23282 PMC5999038 28746310

[36] Fang S Xu C Zhang Y Xue C Yang C Bi H. Umbilical Cord-Derived Mesenchymal Stem Cell-Derived Exosomal MicroRNAs Suppress Myofibroblast Differentiation by Inhibiting the Transforming Growth Factor-β/SMAD2 Pathway During Wound Healing. Stem Cells Trans Med (2016) 5(10):1425–39. doi: 10.5966/sctm.2015-0367 PMC5031180 27388239

[37] Zhu Z Zhang Y Zhang Y Zhang H Liu W Zhang N. Exosomes Derived From Human Umbilical Cord Mesenchymal Stem Cells Accelerate Growth of VK2 Vaginal Epithelial Cells Through MicroRNAs *In Vitro* . Hum Reprod (2019) 34(2):248–60. doi: 10.1093/humrep/dey344 30576496

[38] Ding C Zhu L Shen H Lu J Zou Q Huang C. Exosomal miRNA-17-5p Derived From Human Umbilical Cord Mesenchymal Stem Cells Improves Ovarian Function in Premature Ovarian Insufficiency by Regulating SIRT7. Stem Cells (2020) 38(9):1137–48. doi: 10.1002/stem.3204 32442343

[39] Wang W Liu Y Guo J He H Mi X Chen C. miR-100 Maintains Phenotype of Tumor-Associated Macrophages by Targeting mTOR to Promote Tumor Metastasis *via* Stat5a/IL-1ra Pathway in Mouse Breast Cancer. Oncogenesis (2018) 7(12):97. doi: 10.1038/s41389-018-0106-y 30563983 PMC6299090

[40] Massa M Croce S Campanelli R Abba C Lenta E Valsecchi C. Clinical Applications of Mesenchymal Stem/Stromal Cell Derived Extracellular Vesicles: Therapeutic Potential of an Acellular Product. Diagnostics (Basel) (2020) 10(12):999. doi: 10.3390/diagnostics10120999 PMC7760121 33255416

[41] Bai L Shao H Wang H Zhang Z Su C Dong L. Effects of Mesenchymal Stem Cell-Derived Exosomes on Experimental Autoimmune Uveitis. Sci Rep (2017) 7(1):4323. doi: 10.1038/s41598-017-04559-y 28659587 PMC5489510

[42] Hai B Shigemoto-Kuroda T Zhao Q Lee RH Liu F. Inhibitory Effects of iPSC-MSCs and Their Extracellular Vesicles on the Onset of Sialadenitis in a Mouse Model of Sjogren's Syndrome. Stem Cells Int (2018) 2018:2092315. doi: 10.1155/2018/2092315 29736173 PMC5875028

[43] Kim H Lee MJ Bae EH Ryu JS Kaur G Kim HJ. Comprehensive Molecular Profiles of Functionally Effective MSC-Derived Extracellular Vesicles in Immunomodulation. Mol Ther (2020) 28(7):1628–44. doi: 10.1016/j.ymthe.2020.04.020 PMC7335740 32380062

[44] Ushio A Arakaki R Otsuka K Yamada A Tsunematsu T Kudo Y. CCL22-Producing Resident Macrophages Enhance T Cell Response in Sjogren's Syndrome. Front Immunol (2018) 9:2594. doi: 10.3389/fimmu.2018.02594 30467506 PMC6236111

[45] Wang J Xia J Huang R Hu Y Fan J Shu Q. Mesenchymal Stem Cell-Derived Extracellular Vesicles Alter Disease Outcomes *via* Endorsement of Macrophage Polarization. Stem Cell Res Ther (2020) 11(1):424. doi: 10.1186/s13287-020-01937-8 32993783 PMC7522905

[46] Lo Sicco C Reverberi D Balbi C Ulivi V Principi E Pascucci L. Mesenchymal Stem Cell-Derived Extracellular Vesicles as Mediators of Anti-Inflammatory Effects: Endorsement of Macrophage Polarization. Stem Cells Trans Med (2017) 6(3):1018–28. doi: 10.1002/sctm.16-0363 PMC5442783 28186708

[47] Liao X Sharma N Kapadia F Zhou G Lu Y Hong H. Kruppel-Like Factor 4 Regulates Macrophage Polarization. J Clin Invest (2011) 121(7):2736–49. doi: 10.1172/JCI45444 PMC3223832 21670502

[48] Wang LX Zhang SX Wu HJ Rong XL Guo J. M2b Macrophage Polarization and Its Roles in Diseases. J Leukoc Biol (2019) 106(2):345–58. doi: 10.1002/JLB.3RU1018-378RR PMC7379745 30576000

[49] Hori S Nomura T Sakaguchi S. Control of Regulatory T Cell Development by the Transcription Factor Foxp3 Science (2003) 299(5609):1057–61. doi: 10.1126/science.1079490 12522256

[50] Harrell CR Jovicic N Djonov V Volarevic V. Therapeutic Use of Mesenchymal Stem Cell-Derived Exosomes: From Basic Science to Clinics. Pharmaceutics (2020) 12(5):474. doi: 10.3390/pharmaceutics12050474 PMC7313713 32456070

[51] Nojehdehi S Soudi S Hesampour A Rasouli S Soleimani M Hashemi SM. Immunomodulatory Effects of Mesenchymal Stem Cell-Derived Exosomes on Experimental Type-1 Autoimmune Diabetes. J Cell Biochem (2018) 119(11):9433–43. doi: 10.1002/jcb.27260 30074271

[52] Won HY Hwang ES. Transcriptional Modulation of Regulatory T Cell Development by Novel Regulators NR4As. Arch Pharm Res (2016) 39(11):1530–6. doi: 10.1007/s12272-016-0803-z 27778276

[53] Sekiya T Kashiwagi I Inoue N Morita R Hori S Waldmann H. The Nuclear Orphan Receptor Nr4a2 Induces Foxp3 and Regulates Differentiation of CD4+ T Cells. Nat Commun (2011) 2(1):269. doi: 10.1038/ncomms1272 21468021 PMC3104557

[54] Sun W Wei FQ Li WJ Wei JW Zhong H Wen YH. A Positive-Feedback Loop Between Tumour Infiltrating Activated Treg Cells and Type 2-Skewed Macrophages Is Essential for Progression of Laryngeal Squamous Cell Carcinoma. Br J Cancer (2017) 117(11):1631–43. doi: 10.1038/bjc.2017.329 PMC5729431 28949956

[55] Okeke EB Uzonna JE. The Pivotal Role of Regulatory T Cells in the Regulation of Innate Immune Cells. Front Immunol (2019) 10:680. doi: 10.3389/fimmu.2019.00680 31024539 PMC6465517

[56] Qiu G Zheng G Ge M Wang J Huang R Shu Q. Mesenchymal Stem Cell-Derived Extracellular Vesicles Affect Disease Outcomes *via* Transfer of microRNAs. Stem Cell Res Ther (2018) 9(1):320. doi: 10.1186/s13287-018-1069-9 30463593 PMC6249826

[57] Fang SB Zhang HY Wang C He BX Liu XQ Meng XC. Small Extracellular Vesicles Derived From Human Mesenchymal Stromal Cells Prevent Group 2 Innate Lymphoid Cell-Dominant Allergic Airway Inflammation Through Delivery of miR-146a-5p. J Extracell Vesicles (2020) 9(1):1723260. doi: 10.1080/20013078.2020.1723260 32128074 PMC7034457

[58] Yang R Huang H Cui S Zhou Y Zhang T Zhou YJ. IFN-γ Promoted Exosomes From Mesenchymal Stem Cells to Attenuate Colitis *via* miR-125a and miR-125b Cell Death Dis (2020) 11(7):603. doi: 10.1038/s41419-020-02788-0 32733020 PMC7393506

[59] Pankratz F Hohnloser C Bemtgen X Jaenich C Kreuzaler S Hoefer I. MicroRNA-100 Suppresses Chronic Vascular Inflammation by Stimulation of Endothelial Autophagy. Circ Res (2018) 122(3):417–32. doi: 10.1161/CIRCRESAHA.117.311428 29208678

[60] Aungier SR Ohmori H Clinton M Mabbott NA. MicroRNA-100-5p Indirectly Modulates the Expression of Il6, Ptgs1/2 and Tlr4 mRNA in the Mouse Follicular Dendritic Cell-Like Cell Line, FL-Y. Immunology (2015) 144(1):34–44. doi: 10.1111/imm.12342 24944008 PMC4264908

[61] Li X Fu N Xing Z. MiR-100 Suppresses Inflammatory Activation of Microglia and Neuronal Apoptosis Following Spinal Cord Injury *via* TLR4/NF-κb Pathway Eur Rev Med Pharmacol Sci (2019) 23(20):8713–20. doi: 10.26355/eurrev_201910_19265 31696457

[62] Chen J Chen J Cheng Y Fu Y Zhao H Tang M. Mesenchymal Stem Cell-Derived Exosomes Protect Beta Cells Against Hypoxia-Induced Apoptosis *via* miR-21 by Alleviating ER Stress and Inhibiting P38 MAPK Phosphorylation. Stem Cell Res Ther (2020) 11(1):97. doi: 10.1186/s13287-020-01610-0 32127037 PMC7055095

[63] Wu J Kuang L Chen C Yang J Zeng WN Li T. miR-100-5p-Abundant Exosomes Derived From Infrapatellar Fat Pad MSCs Protect Articular Cartilage and Ameliorate Gait Abnormalities *via* Inhibition of mTOR in Osteoarthritis. Biomaterials (2019) 206:87–100. doi: 10.1016/j.biomaterials.2019.03.022 30927715

[64] Liang C Liu Y Xu H Huang J Shen Y Chen F. Exosomes of Human Umbilical Cord MSCs Protect Against Hypoxia/Reoxygenation-Induced Pyroptosis of Cardiomyocytes *via* the miRNA-100-5p/FOXO3/NLRP3 Pathway. Front Bioeng Biotechnol (2020) 8:615850. doi: 10.3389/fbioe.2020.615850 33520966 PMC7844314

[65] Blokland SLM Hillen MR Wichers CGK Zimmermann M Kruize AA Radstake T. Increased Mtorc1 Activation in Salivary Gland B Cells and T Cells From Patients With Sjogren's Syndrome: mTOR Inhibition as a Novel Therapeutic Strategy to Halt Immunopathology? RMD Open (2019) 5(1):e000701. doi: 10.1136/rmdopen-2018-000701 30713717 PMC6340518

