# Supplementary material for: MSC-Derived Small Extracellular Vesicles Attenuate Autoimmune Dacryoadenitis by Promoting M2 Macrophage Polarization and Inducing Tregs via miR-100-5p
Source: Front Immunol. 2022 Jul 6;13:888949. doi: 10.3389/fimmu.2022.888949 (PMC9298967; doi:10.3389/fimmu.2022.888949)
Supplement: Supplementary file 1 [file DataSheet_1.docx]

Supplementary Material

## Supplementary Figures





**Supplementary Figure 1.** Q-PCR analysis of miR-100-5p levels in LGs collected from rabbits in the untreated and hUC-MSC-sEVs group. n = 6 rabbits per group. Data were shown as mean± SD. **P < 0.01.

**
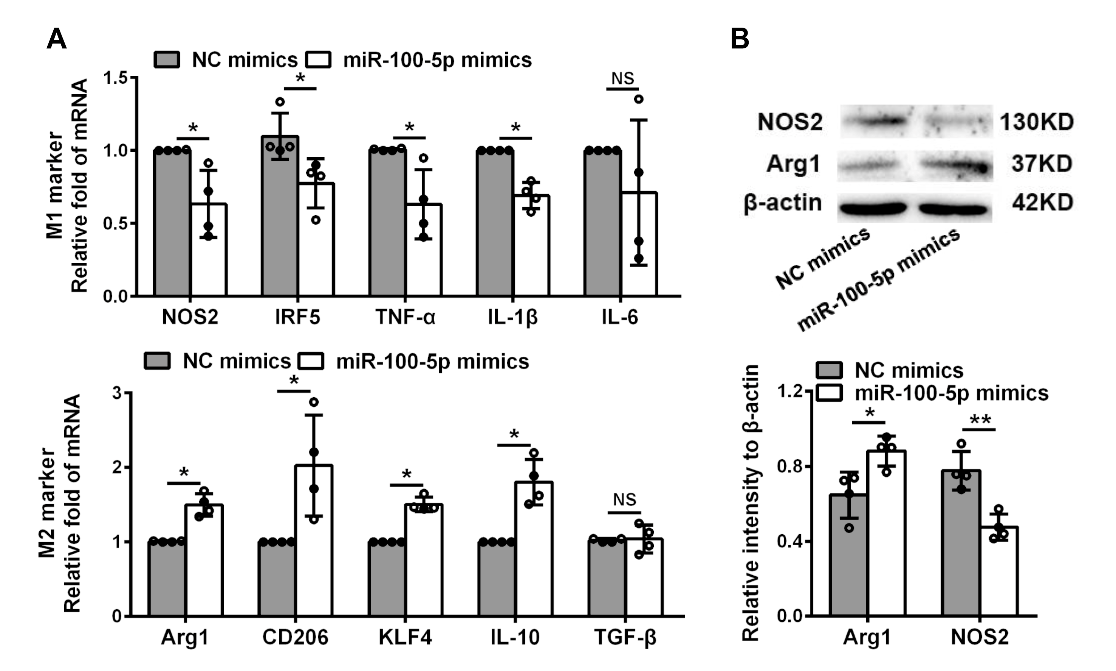
**

**Supplementary Figure 2.** The direct effect of miR-100-5p on macrophage polarization. LPS+IFN-γ-stimulated Mac was transfected with miR-100-5p mimics or NC mimics for 48h. (A) Gene expression profiles of M1 markers (NOS2, IRF5, TNF-α, IL-1β and IL-6) and M2 markers (Arg1, CD206, KLF4, IL-10 and TGF-β). (B) Western blot to assess protein levels of NOS2 and Arg1. Data were from at least three independent experiments and presented as mean± SD. *P < 0.05, **P < 0.01, NS = not significant.


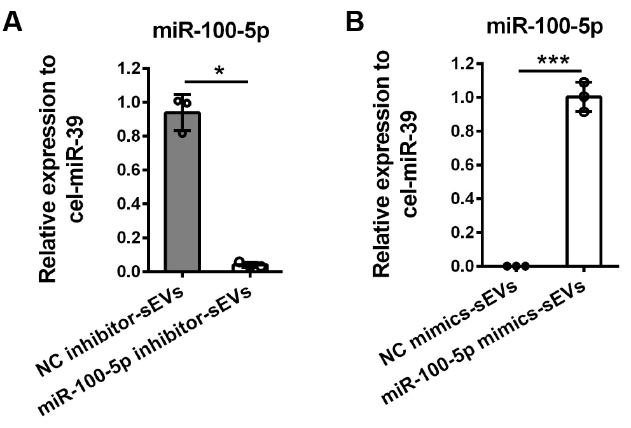


**Supplementary Figure 3.** (A) Q-PCR analysis of miR-100-5p levels in miR-100-5p inhibitor-sEVs and NC inhibitor-sEVs. (B) Q-PCR analysis of miR-100-5p levels in miR-100-5p mimics-sEVs and NC mimics-sEVs. NC: negative-control. Data were from three independent experiments and presented as mean± SD. *P < 0.05, ***P < 0.001.


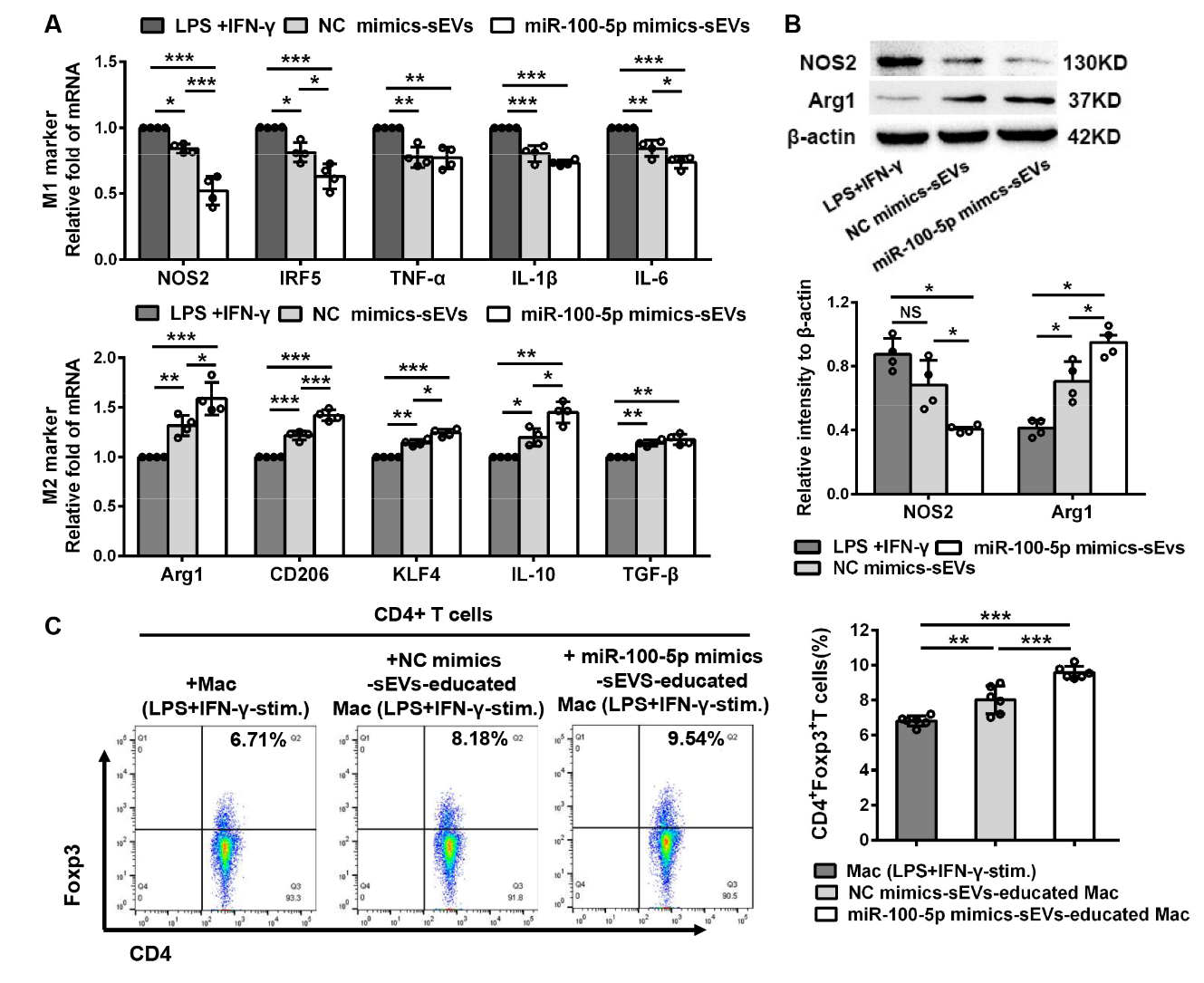


**Supplementary Figure 4.** (A-B) LPS+IFN-γ-stimulated Mac was co-cultured with miR-100-5p mimics-sEVs or NC mimics-sEVs for 48h. (A) Q-PCR analysis of M1 markers (NOS2, IRF5, TNF-α, IL-1β and IL-6) and M2 markers (Arg1, CD206, KLF4, IL-10 and TGF-β). (B) Protein analysis for NOS2 and Arg1. (C) Mac pretreated with miR-100-5p mimics-sEVs or NC mimics-sEVs were co-cultured with human CD4+ T cells stimulated by anti-CD3/-CD28. Flow cytometry analysis was performed for CD4+Foxp3+ T cells. sEVs, hUC-MSC-sEVs; LPS+IFN-γ-stim., LPS+IFN-γ-stimulation. NC: negative-control. Representative data from at least three independent experiments were presented as mean± SD. *P < 0.05, **P < 0.01, ***P < 0.001, NS = not significant.
