# Supplementary material for: MSC-Derived Small Extracellular Vesicles Attenuate Autoimmune Dacryoadenitis by Promoting M2 Macrophage Polarization and Inducing Tregs via miR-100-5p
Source: Front Immunol. 2022 Jul 6;13:888949. doi: 10.3389/fimmu.2022.888949 (PMC9298967; doi:10.3389/fimmu.2022.888949)
Supplement: Supplementary file 2 [file DataSheet_2.docx]

Supplementary Material

## Supplementary Tables

**Supplementary Table 1**. Gene-specific primers used for Q-PCR (rabbit)

| Gene | Forward Primer Seqence | Reverse Primer Sequence |
| --- | --- | --- |
| GAPDH | 5’-GGGTGGTGGACCTCATGGT-3’ | 5’-CGGTGGTTTGAGGGCTCTTA-3’ |
| NOS2 | 5’-TCCACCAGGAGATGCTCAACT-3’ | 5’-TGGGTTTTCCACGCCTCTAC-3’ |
| IRF5 | 5’-CAGTGGGTCAATGGGGAGAG-3’ | 5’-GGCCTTGAAGATGGTGTTGTC-3’ |
| CD206 | 5’-CTGATAGATGGAGGGTGAGGTACA-3’ | 5’-CCAGATAGACGCATGCTGACTTC-3’ |
| Arg1 | 5’-GAAGTAACTCGAACGGTGAACACA-3’ | 5’-TCCCGAGCAACTCCAAAAGA-3’ |
| KLF4 | 5’-ACGACCTCCTGGACCTAGATTTT-3’ | 5’-GGCCACCGACTCCTGATG-3’ |
| Nurr1 | 5’-CTGGCTGTTGGGATGGTTAAA-3’ | 5’-CGACCTCTCCGGCCTTTT-3’ |
| Foxp3 | 5’-CAGCGGACACTCAACGAGATC-3’ | 5’-TTTCGGAAGAAGGCGAACAT-3’ |
| IL-10 | 5’-GGCTGAGGCTGCGACAAT-3’ | 5’-TGCCTTGCTCTTGTTTTCACA-3’ |
| TGF-β | 5’-CAAGGACCTGGGCTGGAA-3’ | 5’-AGGCAGAAGTTGGCGTGGTA-3’ |
| IL-6 | 5’-GCAGAAAAACCAGTGGCTGAA-3’ | 5’-GGCCGCGCAGGATGA-3’ |
| TNF-α | 5’-AGCTTCTCGGGCCCTGAGT-3’ | 5’-CCACTTGCGGGTTTGCTACT-3’ |
| IL-1β | 5’-CTCCTGCCAACCCTACAACAA-3’ | 5’-TCCAGAGCCACAACGACTGA-3’ |

**Supplementary Table 2**. Gene-specific primers used for Q-PCR (human)

| Gene | Forward Primer Seqence | Reverse Primer Sequence |
| --- | --- | --- |
| GAPDH | 5’-CTGGGCTACACTGAGCACC-3’ | 5’-AAGTGGTCGTTGAGGGCAATG-3’ |
| NOS2 | 5’-CCCCTTCAATGGCTGGTACA-3’ | 5’-GCGCTGGACGTCACAGAA-3’ |
| IRF5 | 5’-ATCCAGCGGGAGGTCAAGA-3’ | 5’-GCTCCACCATGCGGTCTTTG-3’ |
| CD206 | 5’-CGCTACTAGGCAATGCCAATG-3’ | 5’-GCAATCTGCGTACCACTTGTTT-3’ |
| Arg1 | 5’-GCGCCAAGTCCAGAACCA-3’ | 5’-CGTGGCTGTCCCTTTGAGAA-3’ |
| KLF4 | 5’-ACCAGGCACTACCGTAAACACA-3’ | 5’-ATGCTCGGTCGCATTTTTG-3’ |
| IL-10 | 5’-TGAGAACAGCTGCACCCACTT-3’ | 5’-TCGGAGATCTCGAAGCATGTTA-3’ |
| TGF-β | 5’-CGCGCATCCTAGACCCTTT-3’ | 5’-CTGTGGCAGGTCGGAGAGA-3’ |
| IL-6 | 5’-AGGGCTCTTCGGCAAATGTA-3’ | 5’GAAGGAATGCCCATTAACAACAA-3’ |
| TNF-α | 5’GCAGGTCTACTTTGGGATCATTG-3’ | 5’-GCGTTTGGGAAGGTTGGA-3’ |
| IL-1β | 5’-TCAGCCAATCTTCATTGCTCAA-3’ | 5’-TGGCGAGCTCAGGTACTTCTG-3’ |

**Supplementary Table 3**. Gene-specific primers used for stem‐loop Q-PCR

| Name | Reverse transcription primer | Forward Primer Seqence | Reverse Primer Sequence |
| --- | --- | --- | --- |
| miR-100-5p | 5’-GTCGTATCCAGTGCAGGGTCCGAGGTATTCGCACTGGATACGACCACAAG-3’ | 5’-GCGAACCCGTAGATCCGAA-3’ | 5’-AGTGCAGGGTCCGAGGTATT-3’ |
| miR-39 | 5’-GTCGTATCCAGTGCAGGGTCCGAGGTATTCGCACTGGATACG-3’ | 5’-GCGTCACCGGGTGTAAATC-3’ | 5’- AGTGCAGGGTCCGAGGTATT-3’ |
| U6 | 5’-TTCACGAATTTGCGTGTCATC-3’ | 5’-CGCTTCGGCAGCACATATAC-3’ | 5’-TTCACGAATTTGCGTGTCATC-3’ |

**Supplementary Table 4**. miRNAs most enriched in the hUC-MSC-sEVs.

| References | Origin | 5 most abundant miRNAs by  high-throughput sequencing |
| --- | --- | --- |
| Fang et al. (36) | hUC-MSC-sEVs vs  293T-sEVs | hsa-miR-21  hsa-miR-125b-1/hsa-miR-125b-2  hsa-miR-23a  **hsa-miR-100**  hsa-let-7f-1/hsa-let-7f-2 |
| Zhu et al. (37) | hUC-MSC-sEVs vs  HDF-sEVs | **hsa-miR-100-5p**  hsa-miR-146a-5p  hsa-miR-21-5p  hsa-miR-221-3p  hsa-miR-143-3p |
| Ding et al. (38) | hUC-MSC-sEVs vs  HDF-sEVs | hsa-miR-199a-5p  **hsa-miR-100-5p**  hsa-miR-224-5p  hsa-miR-17-5P  hsa-miR-370-3p |
